# OrthoReD: a rapid and accurate orthology prediction tool with low computational requirement

**DOI:** 10.1186/s12859-017-1726-5

**Published:** 2017-06-21

**Authors:** Kai Battenberg, Ernest K. Lee, Joanna C. Chiu, Alison M. Berry, Daniel Potter

**Affiliations:** 1 Department of Plant Sciences, University of California, Davis, CA USA; 2 Department of Entomology and Nematology, University of California, Davis, CA USA

**Keywords:** Gene orthology, Phylogenetics, Gene evolution, Genome, Transcriptome

## Abstract

**Background:**

Identifying orthologous genes is an initial step required for phylogenetics, and it is also a common strategy employed in functional genetics to find candidates for functionally equivalent genes across multiple species. At the same time, in silico orthology prediction tools often require large computational resources only available on computing clusters. Here we present OrthoReD, an open-source orthology prediction tool with accuracy comparable to published tools that requires only a desktop computer. The low computational resource requirement of OrthoReD is achieved by repeating orthology searches on one gene of interest at a time, thereby generating a reduced dataset to limit the scope of orthology search for each gene of interest.

**Results:**

The output of OrthoReD was highly similar to the outputs of two other published orthology prediction tools, OrthologID and/or OrthoDB, for the three dataset tested, which represented three phyla with different ranges of species diversity and different number of genomes included. Median CPU time for ortholog prediction per gene by OrthoReD executed on a desktop computer was <15 min even for the largest dataset tested, which included all coding sequences of 100 bacterial species.

**Conclusions:**

With high-throughput sequencing, unprecedented numbers of genes from non-model organisms are available with increasing need for clear information about their orthologies and/or functional equivalents in model organisms. OrthoReD is not only fast and accurate as an orthology prediction tool, but also gives researchers flexibility in the number of genes analyzed at a time, without requiring a high-performance computing cluster.

**Electronic supplementary material:**

The online version of this article (doi:10.1186/s12859-017-1726-5) contains supplementary material, which is available to authorized users.

## Background

As high-throughput sequencing has become more and more accessible, bottlenecks in comparative genetics now generally do not occur at the stage of generation of new sequences, but rather at the stage of downstream analyses that require large computational resources. One of the first steps required for phylogenetic analyses of genome-scale nucleotide or amino acid sequence datasets is determining an orthologous set of genes across multiple species. Orthologs are defined as genes derived from a common ancestral gene that have diverged from one another by a series of speciation events, in contrast to paralogs, which diverge following gene duplication events [1]. There are numerous orthology prediction tools, but most if not all tools share the common initial step of calculating similarity scores using BLAST [2] or BLAST-like algorithms within the sequence dataset. As previously described [3], these tools can be categorized as tree-building-based tools and non-tree-building tools. Tree-building-based tools infer orthology according to the reconstructed phylogeny of a subset of genes showing high similarity among them, e.g., orthology analysis using MCMC [4], OrthologID [5], and PoFF [3], while non-tree-building tools infer orthology directly from the similarity scores, e.g., OrthoMCL [6], HaMStR [7], InParanoid [8], OMA-GETHOGs [9], and bidirectional best hit (BBH) [10, 11].

In general, non-tree-building tools have the advantage of being computationally less demanding i.e. they require fewer CPU (Central Processing Unit) hours and less memory. Particularly in prokaryotes, BBH, the simplest of all non-tree-building methods, can generate reliable results [10, 11], but in plants and animals where gene duplication is more common [12], BBH and other non-tree-building tools can be less accurate [12] due to the presence of multiple genes that are similar to the gene of interest. Tree-building-based tools, on the other hand, are considered more accurate since they are both less sensitive to the effects of sometimes-misleading similarity scores [3, 13] and more consistent with the phylogenetic definition of orthology. At the same time, tree-building-based tools are more computationally demanding. One approach to reduce the computational demand of a tree-building-based tool is to reduce the amount of data handled at one time.

**Fig. 1:**
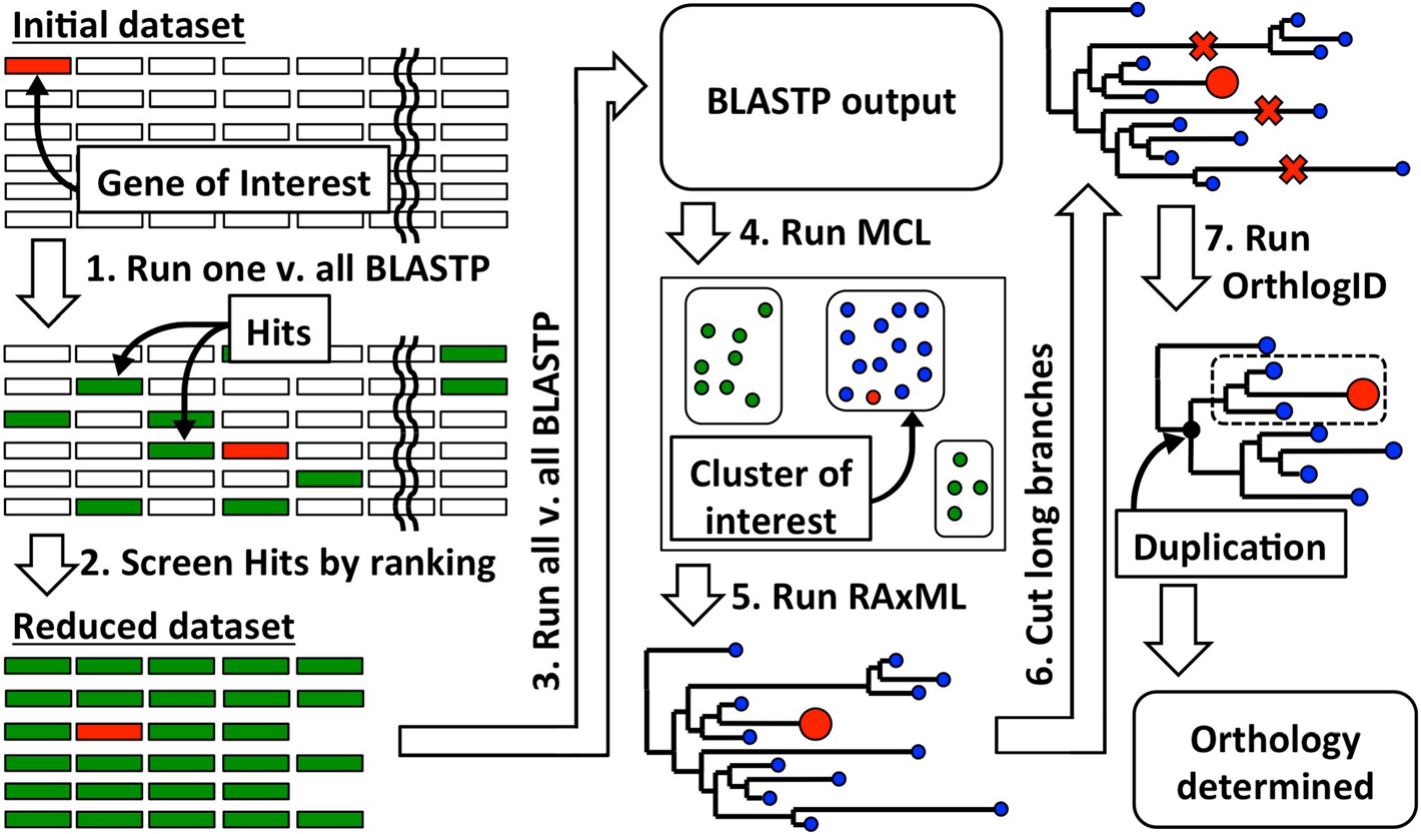
OrthoReD overview. To determine the orthology of the gene of interest, gene of interest is used as a query for a BLASTP search against the dataset (step 1). The BLASTP hits are screened to generate a reduced dataset (step 2). All-v-all BLASTP is conducted on the reduced dataset (step 3) to generate pairwise similarity matrix used by MCL to separate the reduced dataset into clusters (step 4). Most likely phylogeny is reconstructed for the members within the cluster of interest (step 5) and long branches are subsequently removed from the tree (step 6). Finally, all members of the clade that share the most recent gene duplication event are returned as predicted orthologs (step 7)

Here we describe OrthoReD (**Ortho**logy predictions from **Re**duced **D**atasets) (Fig. 1), a tree-building-based orthology prediction tool designed to be executed on desktop computers with accuracy comparable to other published tree-building-based tools. The computational requirement is minimized by 1) generating a reduced dataset only for each gene of interest, and 2) limiting the number of genes that enter the tree-building step.

To test the accuracy of OrthoReD, outputs of OrthoReD were compared with those from two other automated ortholog prediction tools, using three datasets representing fruit flies, plants, and bacteria. The effects of changing parameter settings on the three most time-consuming processes (BLAST, sequence alignment, and tree-building) were also assessed to determine the optimal balance between speed and accuracy. In addition, the runtime for each of these parameter adjustments was measured to assess the impact of these adjustments not only on the output but also on the speed of the analysis.

## Implementation

### OrthoReD overview

The framework of OrthoReD is a basic Perl script that utilizes published and/or commonly used bioinformatic tools. The program only requires an initial gene dataset (as one or more FASTA files) with all genes labeled with their species and a unique ID, but information about isoforms (splice variants) and the outgroup species within the dataset can also be provided.

From within the initial dataset, OrthoReD searches for orthologs for each gene of interest at a time (one-at-a-time approach) rather than grouping all genes into orthologous sets at once (all-at-once approach), as seen for example in OrthologID [5] or OrthoMCL [6] (Step 1 in Fig. 1). Gene sequence(s) of interest is(are) provided as a single query file in FASTA format. All following steps are conducted only within the reduced dataset generated according to the initial one-against-all comparison. This feature of reducing the dataset for the downstream analysis is similar to the pipeline used for building the phylogeny repository PhylomeDB [14, 15]. However, the purpose of this step is different in the two pipelines: the pipeline for PhylomeDB performs this process on a selected subset of species within the dataset to define the phylogenetic scope and calibrate itself for the downstream analysis. OrthoReD uses all species within the dataset and directly uses the outcome for orthology predictions.

Breaking down the enormous task of predicting all orthologies within the initial dataset to a single gene at a time allows OrthoReD to be executed on a desktop computer and, more importantly, this allows the flexibility of predicting orthology for any subset of genes within the dataset that are particularly interesting to the user without analyzing the entire dataset.

By default, OrthoReD conducts BLASTP searches by using NCBI BLAST v2.40 [2] with parameter settings optimized for orthology predictions with soft masking and Smith-Waterman final alignment (parameter settings: -db_soft_mask 21 -word_size 3 -threshold 11 -evalue 1e-03 -use_sw_tback) [16]. Alternatively, similarity searches can be conducted using AB BLAST v3.0 [17] or SWIPE v2.0.12 [18]. The default parameters for AB BLASTP are optimized for less sensitive but faster searches (parameter settings: wordmask = seg matrix = BLOSUM62 E = 1e-3 W = 3 *T* = 1000 postsw) according to [19]. AB BLAST is implemented as an alternative method because AB BLAST, compared to NCBI BLAST, generally allows the user more flexibility in setting search parameters. (Simply increasing T or W will drastically improve runtime of AB BLASTP). SWIPE is also implemented as another alternative for potentially faster and more accurate similarity search option (parameter settings: --evalue = 1e-03 --symtype = blastp).

The genes with e-values better than the minimum threshold (default: <1e-3) in comparisons with the gene of interest (“BLAST or SWIPE hits”, henceforth hits) are screened further (Step 2 in Fig. 1). By default, a given hit is retained if it meets the minimum level of sequence similarity, which is calculated based on alignment length and % sequence identity [20]. Alternatively, hits can also be screened based on a user-defined minimum threshold for alignment length and/or % identity.

Then, more importantly, an additional parameter, *n*, is set to limit the number of genes per species passed on to the next step. Although the similarity score between a particular gene and the gene of interest is generally correlated with likelihood that it is an ortholog, that particular ortholog may not show the highest similarity or have the best e-value within the genome of another species [12, 21]. On the other hand, similarity scores and phylogenetic relatedness should broadly correlate [21]. Thus, it is unlikely that a given genome will have a large number of non-orthologous genes (paralogs) that show higher similarities to the gene of interest than the ortholog. Therefore, a parameter is implemented that will keep only a number of gene sequences up to *n* (an integer defined by the user) best hits per species in terms of BLAST e-values (all genes are kept upon a tie). This permits screening of sequences that should be analyzed further as potential orthologs. The optimal *n* is gene-specific, but in practice a single *n* is set for each query file. A query file can contain one or many genes, each with potentially different optimal *n*. Tests for optimizing *n* for queries with multiple genes are presented in Results and strategies further discussed in the Discussion. When isoform information for genes is available, only one isoform with the best e-value is kept as a representative of the gene.

Next, an all-against-all BLASTP search with the same parameter settings as the initial similarity search is conducted on the reduced dataset to generate a pairwise similarity score matrix (Step 3 in Fig. 1). For any pair within the reduced dataset without a significant level of similarity (e-value <1e-3), a very large value (e-value = 9e-1) is set in order to ensure that all pairs will have a value assigned.

Because *n* (the maximum number of genes per species allowed into the tree-building step) is a fixed threshold applied to all species in the dataset, it is possible for genes that are highly unlikely to be orthologous to be present in the reduced dataset. The genes in the reduced dataset are therefore separated into clusters using MCL v14–137 [22] to remove such outliers based on the pairwise similarity score matrix generated in the previous step (Step 4 in Fig. 1). That being said, because a gene tree is a more accurate criterion for ortholog prediction than similarity scores, the inflation rate of MCL, the parameter that determines the inclusiveness of clusters, by default is set to be the least stringent (parameter settings: *i* = 1.2) to minimize the risk of erroneously removing orthologs at this step.

To prepare for tree-building (Step 5 in Fig. 1), a multiple sequence alignment (MSA) is generated with all genes in the cluster of interest using MAFFT v7.273 [23] with the accuracy-oriented parameter setting (parameter settings: --localpair --retree 2 --maxiterate 1000) by default. However, the speed-oriented parameter setting (parameter settings: --6merpair --retree 2 --maxiterate 1000) is used instead when 200 or more sequences are aligned as recommended by MAFFT. The quality of the MSA is ensured by limiting the sequences given to MAFFT to a small number (less than *n* times the number of species included in the dataset) of highly similar sequences, which also ensures a quick sequence alignment. Based on this MSA, the most likely gene tree is reconstructed using RAxML v8.2.8 [24]. To generate the gene tree as quickly as possible, the topology of the tree is determined first (parameter settings: -f E -F -m PROTCATIAUTO), and the optimal branch lengths are estimated subsequently (parameter settings: -f e -m PROTCATIAUTO). The tree is then rooted by a gene of a user-defined outgroup. When multiple genes of an outgroup are present in the tree, then it is rooted using the outgroup gene that results in a sub-tree that includes the gene of interest, but no outgroup gene, and with the largest number of ingroup species (RT method [13]). The most distant outgroup gene based on branch length is selected upon a tie. In the highly unlikely event that the two or more outgroup genes are equally distant from the gene of interest, it is selected based on alphanumeric order. When an outgroup is not provided or no outgroup gene is present in the gene tree (e.g. due to gene loss or missing data), the tree is rooted at midpoint. Because midpoint rooting may not orient the tree such that the orthologs of the gene of interest form a single clade, it is recommended to use an outgroup for better accuracy.

Next, any long external and internal branches (default: >2 substitution/site) in the generated tree are cut (Step 6 in Fig. 1) because the accuracy of the positioning of these long branches can be difficult to assess for both internal [25] and external [13] branches, a difficulty described in other ortholog identification pipelines [13].

Finally, the orthologous set of genes for the gene of interest is determined by implementing a part of “Diagnostics Generator module” of OrthologID [5] (Step 7 in Fig. 1). This final step determines whether each node in the gene tree is a speciation event or a gene duplication event. Traversing the tree from root to tip, at each node it is tested to determine if there is an overlap in the species represented by the genes in each of the sister clades under the node. If any overlap is present, the node is considered a gene duplication event, unless all genes under the node are representing a single species, in which case, the entire clade defined by this node is treated as a single gene with multiple isoforms. This approach accounts for datasets where isoforms are included without being labeled as such. When a given node is determined to be a gene duplication event, all of its ancestral nodes previously determined as speciation events are overwritten as gene duplication events. Once the status of all nodes is determined, all members of the daughter clade of the most recent gene duplication event experienced by the gene of interest that includes the gene of interest is returned as a set of predicted orthologs. Only the members of this clade are returned as orthologs while others in the tree are discarded. When isoform information for genes is available, all other isoforms of the predicted orthologs within the original dataset are reintroduced as predicted orthologs as well.

### Experimental datasets

Three datasets (FLY, PLANT, and ACTINO) were generated in order to assess the outputs and runtimes of OrthoReD under different parameter settings (Table 1, Additional file 1: Table S1). All species included in FLY were within the genus *Drosophila* except for the outgroup, *Lutzomyia longipalpis*. Sequences for FLY were obtained from OrthoDB [26, 27] and included all amino acid sequences from 13 closely related species. All species included in PLANT were within Rosids, a clade within Angiosperms. *Vitis vinifera*, the most early-divergent among the 12 species according to APG III [28], was selected as the outgroup. Sequences for PLANT were obtained from Phytozome v11.0 [29, 30] and included all coding sequences (CDSs) from 12 species. All species included in ACTINO were within phylum Actinobacteria. *Eggerthella lenta* was selected as the outgroup [31]. Sequences for ACTINO were obtained from IMG [32, 33] and included all CDSs from 100 species selected according to Sen et al. [31].

**Table 1:** Dataset information

	Fly	Plant	Actino
Number of taxa	13	11	100
Lis of taxa	*Drosophila ananassae*, *D. erecta*, *D. grimshawi*, *D. melanogaster*, *D. mojavensis*, *D. persimilis*, D. pseudoobscur, D. sechellia, *D. simulans*, *D. virilis*, *D. willistoni*, D. yakuba, *Lutzomyia longipalpis*	*Arabidopsis thaliana*, *Cucumis sativus*, *Fragaria vesca*, *Glycine max*, *Mallus domestica*, *Manihot esculenta*, *Medicago truncatula*, *Phaseolus vulgaris*, *Populus trichocarpa*, *Prunus persica*, *Vitis vinifera*	See supplemental Table S1
Range of taxa	Genus *(Drosophila*)	Rosids	Phylum (Actinobacteria)
Selected outgroup	*Lutzomyia longipalpis*	*Vitis vinifera*	*Eggerthella lenta*
Number of sequences	194,469	532,305	444,382
Number of AA residues	92,515,839	215,684,745	146,754,746
Average sequence length	476	405	330

These three dataset differ significantly in their overall composition. Orthology prediction was expected to be most straightforward for FLY, containing only closely related species. FLY was designed to test 1) how consistent the output of OrthoReD is as compared to other ortholog prediction tools and 2) how changing parameter settings for MSA building affects the output of OrthoReD. PLANT, while having similar number of species as FLY, had two aspects that should make orthology prediction more complex than in FLY: First, the species included were much more distantly related from each other than in FLY, ranging across seven orders. Secondly, plant genomes are known for high levels of gene duplication [34]. PLANT was designed to test 1) how consistent the output of OrthoReD is as compared to other ortholog prediction tools and 2) how changing parameter settings for BLAST and *n* each affects the output of OrthoReD. ACTINO includes more than six times as many species as FLY or PLANT, and was designed to test the impact on the runtime of OrthoReD of a substantially larger number of species.

All nucleotide sequences of PLANT and ACTINO were translated into amino acid sequences using a custom Perl script. A small fraction of the nucleotide sequences originally collected for the PLANT and ACTINO datasets (<0.05% and <0.3%, respectively) could not be translated reliably due to ambiguous reading frames or premature stop codons. These sequences were removed from the analyses.

### Comparison of OrthoReD runtime and outputs

OrthoReD can be executed under different conditions by adjusting parameter settings for each step. In general, adjustments to improve accuracy will have the tradeoff of reduced speed of orthology prediction. Therefore, for efficient orthology prediction, the parameters need to be set to achieve the maximum speed possible without a significant loss of accuracy. The three most time-consuming steps in OrthoReD (as in many tree-building-based tools) in the order they appear in OrthoReD are (1) the initial similarity search, (2) the generation of the MSA (particularly in cases where one or more sequence within the cluster of interest was exceptionally long), and (3) the tree-building step. Therefore, for each dataset, OrthoReD was executed multiple times using different parameter settings for similarity search (BLAST or SWIPE), MAFFT, and *n* to compare the outputs and runtimes to assess the impact of each adjustment.

The outputs for OrthoReD were also compared with that of two other published orthology prediction tool: OrthologID [5] and OrthoDB [26]. These two tools were chosen based on their degree of similarity to OrthoReD. OrthologID is a tree-building-based all-at-once approach tool that shares the common final orthology determination step with OrthoReD. On the other hand, OrthoDB is an example of non-tree-building all-at-once approach tools which also include OrthoMCL [6] and HaMStR [7]. OrthologID and OrthoDB thus represent very different orthology prediction methods. For each dataset, only one output generated under the default parameter settings was used for OrthologID and OrthoDB.

For the FLY dataset, orthologs were predicted for all *Drosophila melanogaster* genes (13,972 genes) under four different conditions: ReD_s.aln with the default conditions of OrthoReD (see section above, *OrthoReD overview*), ReD_f.aln for speed-oriented sequence alignment, OID (OrthologID with default conditions), and ODB (OrthoDB with default conditions) (see Table 2 for parameter settings for each OrthoReD execution). OrthoDB provided predicted orthology for all genes on their website [27].

**Table 2:** Parameter settings for each OrthoReD execution on each dataset

	Dataset	Similarity search	n*	MAFFT options
ReD_s.aln	FLY	NCBI	4	--localpair --retree 2 --maxiterate 1000
ReD_f.aln	FLY	NCBI	4	--6merpair --retree 2 --maxiterate 1000
ReD_AB	PLANT	AB	5	--6merpair --retree 2 --maxiterate 1000
ReD_SW	PLANT	SWIPE	5	--6merpair --retree 2 --maxiterate 1000
ReD_n5	PLANT	NCBI	5	--6merpair --retree 2 --maxiterate 1000
ReD_n6	PLANT	NCBI	6	--6merpair --retree 2 --maxiterate 1000
ReD_n7	PLANT	NCBI	7	--6merpair --retree 2 --maxiterate 1000
ReD_n8	PLANT	NCBI	8	--6merpair --retree 2 --maxiterate 1000
ReD_n9	PLANT	NCBI	9	--6merpair --retree 2 --maxiterate 1000
ReD_n10	PLANT	NCBI	10	--6merpair --retree 2 --maxiterate 1000
ReD_n4	ACTINO	NCBI	4	--6merpair --retree 2 --maxiterate 1000

*n: Maximum number of genes per species passed on after the initial similarity search

For PLANT, orthologs were predicted for all *Arabidopsis thaliana* genes (27,412 genes) under nine different conditions: ReD_n5 through ReD_n10 with speed-oriented MAFFT setting and *n* ranging from 5 to 10; ReD_AB and ReD_SW using AB BLAST or SWIPE instead of NCBI BLAST; and OID (OrthologID with default conditions) (Table 2). Isoform information was available for each *A. thaliana* CDS (35,382 CDSs were available for the 27,412 genes). For a gene with multiple isoforms, OrthoReD predicted orthology for all isoforms, and the union of the predicted sets of all isoforms was considered the predicted set of orthologs for one gene. OrthologID used the longest isoform as a representative to predict orthology for the gene.

For ACTINO, orthologs were predicted for all *Streptomyces coelicolor* genes (8210 genes) under a single condition: ReD_n4 (Table 2).

For a single gene of interest, the similarity between the outputs under two different conditions was assessed by the % identity of the two sets of predicted orthologs, calculated as the number of genes in the intersection over the number of genes in the union. OrthoReD uses an outgroup to root the gene tree (Step 5 in Fig. 1) making it unsuitable for predicting orthologs of the outgroup. Therefore, any genes of the selected outgroup were removed from the output comparison. The overall similarity of outputs between different conditions was assessed based on the average % identity of all genes tested, or % genes with % identity above a threshold (> = 90% or =100%).

Because the runtime on a computing cluster is highly dependent on how heavily the cluster is used, the runtime can be inconsistent from one instance to another. Therefore, for each execution of OrthoReD in the output comparison, an independent test of OrthoReD was conducted in parallel with identical parameter settings to measure the runtime. For this, 1000 *D. melanogaster*, *A. thaliana*, or *S. coelicolor* genes were randomly selected without replacement for each dataset. For each gene, the runtime was measured as total CPU time, using the UNIX time command. Total CPU time was calculated as the sum of user and system CPU time. This runtime test was conducted on a Mac Pro with OS X El Capitan v10.11.6 operating system, with 3.33GHz 6-Core Intel Xeon processor. Each runtime test was allocated 2 cores for processing.

### Testing orthology predictions for optimal *n* for each dataset

For each of the conditions for orthology prediction tested in FLY, PLANT and ACTINO, the total number of orthologs predicted was counted and the distribution of predicted orthologs according to the initial BLASTP (or SWIPE) e-values (Step 1 in Fig. 1) per species (genome) was calculated. For example, a predicted ortholog was ranked 1 when this gene had the best e-value within the genome, whereas if there were three other genes with better e-values the ortholog was ranked 4. NCBI BLASTP e-values generated by Step 1 of OrthoReD were used for ranking outputs of OID and ODB. Along with the ranking distribution, the total number of predicted orthologs was also counted.

### Merging of predicted orthologous groups

Because OrthoReD predicts orthology for each gene of interest independently, predicted orthologous groups are not mutually exclusive. Thus, upon predicting orthologies of multiple genes within the dataset, it is possible that a given gene may be predicted as an ortholog of more than one gene of interest.

To assess the degree of overlap across orthologous groups predicted in each dataset, predicted orthologous groups for ReD_s.aln on FLY, ReD_n10 on PLANT, and ReD_n4 on ACTINO were each merged into non-overlapping groups. We then measured the fraction of merged groups that were identical to at least one of the predicted orthologous groups, and measured the fraction of genes of interest that were within such merged groups.

## Results

### Comparison of outputs across different conditions

**Fig. 2:**
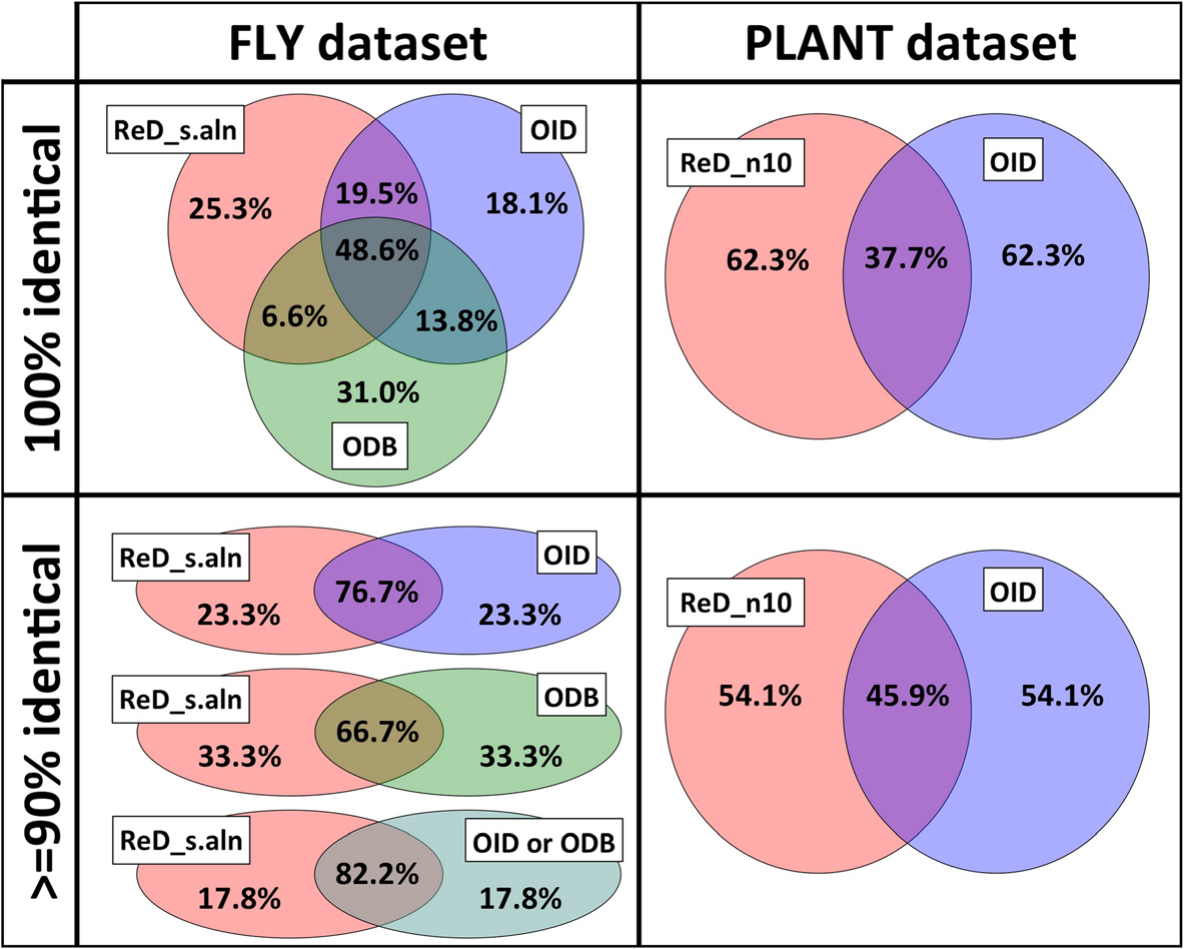
Output comparison between OrthoReD, OrthologID, and OrthoDB. The overall similarities of the outputs in two datasets (FLY and PLANT) generated under different conditions are compared based on the fraction of genes of interest with % identity above a threshold. ReD_s.aln and ReD_n10 (*red*) used OrthoReD, OID (*blue*) used OrthologID, and ODB (*green*) used OrthoDB to generate the output

For the 13,972 genes of interest tested from the FLY dataset, ReD_s.aln, ReD_f.aln, OID, and ODB predicted a total of 156,223, 157,105 142,437, and 219,715 orthologs respectively (Table 3). Outputs of ReD_s.aln and ReD_f.aln were 96.4% identical on average, and 89.5% of the genes had 100% output identity (Table 4). Outputs of ReD_s.aln and OID were 88.3% identical on average and 68.6% of the genes had 100% output identity (Table 4). Outputs of OID and ODB were 80.5% identical on average and 56.2% of the genes had 100% output identity (Table 4). ReD_s.aln, generated output with 100% identity to either OID or ODB for 74.7% of the genes tested, and with > = 90% identity for 82.2% of the genes tested (Fig. 2).

**Table 3:** Total count of predicted orthologs and distribution of predicted orthologs at each e-value rank under different conditions of ortholog prediction

	Fly				Plant									Actino
	ReD_s.aln	ReD_f.aln	OID	ODB	ReD_AB	ReD_SW	ReD_n5	ReD_n6	ReD_n7	ReD_n8	ReD_n9	ReD_n10	OID	ReD_n4
Total count	156,223	157,105	142,437	219,715	489,574	424,334	514,093	519,680	524,341	531,003	534,538	537,454	295,966	169,860
1	94.0%	94.0%	95.4%	79.3%	76.6%	69.6%	75.8%	74.5%	73.4%	72.7%	72.2%	71.6%	66.6%	92.8%
2	3.9%	3.9%	2.7%	7.2%	10.7%	14.5%	10.7%	10.5%	10.3%	10.1%	10.0%	9.9%	8.7%	5.1%
3	1.3%	1.3%	0.7%	3.4%	6.3%	7.8%	6.4%	6.2%	6.0%	5.9%	5.8%	5.8%	4.6%	1.4%
4	0.8%	0.8%	0.3%	1.9%	3.9%	4.9%	4.2%	4.0%	3.9%	3.8%	3.7%	3.7%	2.8%	0.7%
5	0.0%	0.0%	0.2%	1.3%	2.5%	3.3%	2.9%	2.8%	2.7%	2.6%	2.5%	2.5%	1.8%	0.0%
6	0.0%	0.0%	0.1%	0.9%	0.0%	0.0%	0.0%	2.1%	2.0%	1.9%	1.9%	1.9%	1.3%	0.0%
7	0.0%	0.0%	0.1%	0.8%	0.0%	0.0%	0.0%	0.0%	1.6%	1.6%	1.6%	1.5%	1.0%	0.0%
8	0.0%	0.0%	0.1%	0.6%	0.0%	0.0%	0.0%	0.0%	0.0%	1.3%	1.3%	1.2%	0.8%	0.0%
9	0.0%	0.0%	0.1%	0.5%	0.0%	0.0%	0.0%	0.0%	0.0%	0.0%	1.0%	1.0%	0.7%	0.0%
10	0.0%	0.0%	0.1%	0.4%	0.0%	0.0%	0.0%	0.0%	0.0%	0.0%	0.0%	0.9%	0.6%	0.0%
11+	0.0%	0.0%	0.4%	3.6%	0.0%	0.0%	0.0%	0.0%	0.0%	0.0%	0.0%	0.0%	11.0%	0.0%

**Table 4:** Comparisons between outputs generated by different conditions of OrthoReD, OrthologID, and OrthoDB

Database		FLY					PLANT															
Conditions Compared	ReD_s.aln	ReD_s.aln	ReD_s.aln	ReD_f.aln	ReD_f.aln	OID	ReD_AB	ReD_SW	ReD_n5	ReD_n6	ReD_n7	ReD_n8	ReD_n9	ReD_n10	ReD_n5	ReD_n5	ReD_AB	ReD_n5	ReD_n6	ReD_n7	ReD_n8	ReD_n9
	ReD_f.aln	OID	ODB	OID	ODB	ODB	OID	OID	OID	OID	OID	OID	OID	OID	ReD_AB	ReD_SW	ReD_SW	ReD_n6	ReD_n7	ReD_n8	ReD_n9	ReD_n10
average % id	96.4%	88.1%	81.2%	88.2%	81.4%	80.5%	58.7%	59.8%	60.5%	61.1%	61.2%	61.3%	61.6%	61.8%	77.9%	72.2%	71.0%	82.3%	85.8%	85.7%	86.1%	87.3%
% 100% id	89.5%	68.1%	55.1%	68.3%	55.1%	56.2%	33.9%	36.1%	35.4%	36.4%	36.8%	36.9%	37.4%	37.7%	58.1%	53.2%	51.3%	62.0%	69.0%	70.0%	71.0%	80.6%
% > =90% id	92.2%	76.7%	66.7%	76.8%	67.0%	67.1%	41.2%	42.6%	43.3%	44.4%	44.8%	44.9%	45.5%	45.9%	65.4%	59.3%	57.8%	72.2%	77.8%	77.8%	78.8%	87.3%

For PLANT, outputs of ReD_n5–10 became more similar to one another as *n* was increased: average % identity between ReD_n5 and ReD_n6 was 82.3% but this steadily increased up to 87.3% between ReD_n9 and ReD_n10 (Table 4). The outputs of ReD_n5–10 also became more similar to OID as *n* increased: Average % identity was 60.5% for ReD_n5 and this steadily increased up to 61.8% for ReD_n10. In general, however, the outputs were less similar between OrthoReD and OrthologID in PLANT than in FLY.

Between the three conditions with different similarity searches, the average % identity was 77.9% between ReD_n5 (NCBI BLAST) and ReD_AB (AB BLAST), 72.2% between ReD_n5 and Red_SW (SWIPE), and 71.0% between ReD_AB and ReD_SW (Table 4). In respect to OID the average % identity for ReD_n5, ReD_AB, and ReD_SW was 60.5%, 58.7%, and 59.8% respectively.

### Comparison of runtimes across different conditions

**Fig. 3:**
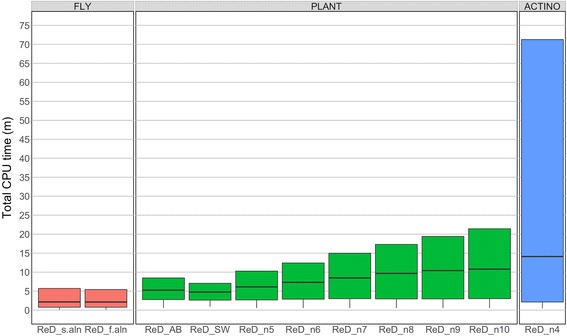
Total CPU time for each condition of OrthoReD. Each box indicates the total CPU time incurred by different conditions of OrthoReD. The line in the box indicates the median, upper and the lower ends of the box indicate the upper and the lower quartiles. The minimum runtime is indicated by the lowest point on the line extended below the box (lowest quartile). The maximum runtime is not indicated

The minimum and median total CPU times were <1 min and <15 min, respectively, under all conditions tested (Fig. 3). The maximum time (not shown on Fig. 3) varied from one condition to another but was <150 min in FLY, <210 min in PLANT, and <790 min in ACTINO. Although wall-clock time (the actual time it takes for OrthoReD to run to completion) varied depending on the overall computer usage, the median wall-clock real time was always <12 min.

Different aligning parameters to MSA generation (ReD_s.aln and ReD_f.aln) generated nearly identical results with <0.3 min difference at minimum, lower quartile, median, and upper quartile. Median time of ReD_AB and ReD_SW were each 14.0% and 22.2% shorter than ReD_n5 with the same *n*. Among the conditions that differed in *n* (ReD_n5–10), the total CPU time steadily increased as *n* increased: For each addition of *n*, the median total CPU time increased between 3.7–19.7%, and between ReD_n5 and ReD_n10 the median time increased by 76.4%.

### Distribution of orthologs at each e-value rank under different thresholds of n

For FLY, 99.2% of the predicted orthologs had e-values that were ranked between 1(best e-value in the genome in based on initial BLAST search with the gene of interest) – and 3 (three other genes had better e-values) by both ReD_s.aln and ReD_f.aln, and only 0.8% of the predicted orthologs were ranked 4 (Table 3). The fraction of orthologs predicted under OID and ODB ranked between 1 and 3 were 98.8% and 90.0% respectively (Table 3).

For PLANT, ReD_n5 through ReD_n10 showed that the total number of genes identified steadily increased as *n* was increased. On the other hand, fractions of orthologs represented by the lowest rank steadily decreased from 2.9% at rank 5 for ReD_n5 to 0.9% at rank 10 for ReD_n10. In both cases, the rate of change became smaller as *n* increased. For instance, the lowest rank representation dropped from 2.9% to 1.3% between ReD_n5 and ReD_n8, but only reached 0.9% at ReD_n10 (Table 3). 11.0% of the orthologs predicted by OID was ranked 11 or lower (Table 3).

For ACTINO, 92.8% of the predicted orthologs were ranked 1, and 99.1% of the predicted orthologs were ranked between 1 and 3 (Table 3).

### Degree of overlap among orthologous groups

The degree of overlap among the predicted orthologous groups generally correlated with the expected difficulty of orthology prediction: FLY, with only closely-related species, had the highest fraction of genes belonging to merged groups that were identical to at least one orthologous group (89.0%); ACTINO, with a larger number of more distantly related taxa, showed a lower fraction (82.1%); and PLANT, with distantly-related taxa with known high degree of gene/genome duplications, showed the lowest (68.4%) (Table 5). Over 90% of the non-overlapping merged groups were identical to at least one orthologous group in all conditions even in ReD_n10 on PLANT.

**Table 5:** The degree of overlap among the predicted orthologous groups under each condition

	Number of genes	Number of merged groups	% Identical to Orthologous group^1^	% Genes rescued^2^
FLY_ReD_s.aln	13,972	12,923	95.1%	89.0%
PLANT_ReD_n10	27,412	16,531	90.4%	68.4%
ACTINO_ReD_n4	8210	6933	92.7%	82.1%

^1^Fraction of merged groups that are identical to at least one orthologous group predicted from one gene of interest

^2^Fraction of genes of interest that belonged to the merged groups in C

## Discussion

### OrthoReD generates output comparable to published tools.

The accuracy of results of any orthology prediction tool ideally would be assessed based on the degree of similarity to the “correct answer” i.e. the true phylogenetic trees for the genes of interest. Since the true trees are unknown, we assessed the accuracy of OrthoReD based on the degree of similarity of results to two other commonly used orthology prediction tools, OrthoDB and OrthologID.

OrthoDB was less stringent than OrthologID or OrthoReD (Table 3), predicting >40% more genes as orthologs than the other two tools tested. The higher average % output identity and fraction of outputs with > = 90% identity indicated that the output of OrthoReD was more similar to that of OrthologID than to that of OrthoDB. Moreover, while outputs of OrthologID and OrthoDB had a higher fraction of identical output by 1.1%, outputs of OrthoReD and OrthoDB had a higher average % identity by 0.7%. Furthermore, OrthoReD and OrthologID generated the most similar pair of outputs. These results indicated that 1) OrthoReD generates results of high similarity to OrthologID, and 2) when OrthoReD predicts a given gene as an ortholog but OrthologID does not, these genes are usually predicted as orthologs by OrthoDB.

The highest average % identity of outputs by OrthoReD and OrthologID in the PLANT was 61.8%, which was substantially lower than 88.1% in the FLY. These findings reflect the substantial difference in the compositions of the two datasets: While FLY only included species from the same genus except for the outgroup, PLANT included species from across seven orders. With greater degree of divergence among the included species, it is expected that different orthology prediction tools will become less consistent. Furthermore, plants are particularly known for their high rate of gene duplication [34], which leads to gene families with relatively high sequence similarity among the members, and makes orthology prediction more difficult. This phenomenon was indicated in our tests by a relatively large fraction of predicted orthologs with a low e-value ranking (Table 3).

### Optimization of *n*

Optimization of the value of *n* requires balancing loss of efficiency (*n* too high) with the risk of false negatives in orthology prediction (*n* too low). As found in the outputs of ReD_n5–10 on PLANT, lower *n* resulted in fewer discoveries of orthologs along with lower similarity to OID suggesting lower accuracy. On the other hand, increasing *n* resulted in increased runtime, and can result in less accurate MSA, which leads to less accurate orthology prediction.

We would expect that the exclusion of true orthologs by reducing the value of *n* could lead to false negatives. At the same time, reducing the value of *n* could also alter the topology of the resulting tree, which might lead to false positives. To address the impact of false positives relative to the impact of false negatives due to lowering the value of *n*, we counted the number of false positives/negative orthologous gene predictions of ReD_n5–10 against OID on PLANT. We found that, as *n* decreases, the number of false negatives increased whereas false positives decreased (Additional file 2: Table S2). Thus the primary risk of lowering the value of *n* is false negatives rather than false positives.

In the cases of ReD_s.aln in the FLY, ReD_n10 in the PLANT, and ReD_n4 in the ACTINO, the lowest rank only represented <1%. Since it is unlikely that further increasing *n* will result in a major improvement in finding otherwise missed orthologs 4, 10, and 4 would be the optimal settings for *n* in the FLY, the PLANT, and the ACTINO respectively.

The difference in optimal *n* among the different datasets was consistent with the different degrees of prevalence of gene duplications among the organisms included in the dataset. ACTINO with bacterial species had the optimal *n* of 4 and >92% of the predicted orthologs ranked 1, and FLY with only closely related species also had the optimal *n* of 4 and >93% of the predicted orthologs ranked 1. On the other hand, PLANT, the dataset with the highest expected degree of gene duplication [34], had the largest optimum *n*.

### Impact of adjusting BLASTP, MSA, and *n* on accuracy and time

The similarity search parameters (NCBI BLAST, AB BLAST, and SWIPE) can have a significant effect on runtime particularly on larger datasets. In OrthoReD, similarity search is implemented as the initial preliminary screening, and as the basis for MCL clustering. In theory, neither of these processes is particularly sensitive to the absolute values of the similarity searches, and our results confirmed this. We found that the average % identity of ReD_n5, ReD_AB, and ReD_SW with respect to OrthologID ranged only by 1.8% (58.7–60.5%). Considering the 22.2% shorter runtime of ReD_SW compared to ReD_n5, the advantage of using SWIPE as the similarity search tool becomes more significant as the dataset becomes larger.

On the other hand, adjustments on the MAFFT parameters only had a marginal effect, as shown by the observation that ReD_s.aln and ReD_f.aln had very similar outputs and runtimes. The output of ReD_f.aln was slightly (0.2% average % identity) more similar to both OID and ODB than ReD_s.aln, but this might occur because OrthologID uses speed-oriented MAFFT for aligning over 500 sequences at once. In any case, since the accuracy-oriented parameter setting is only recommended with <200 sequences, it is unlikely that using speed-oriented parameter settings of ReD_f.aln in all cases will be preferred over ReD_s.aln under any scenario.

### Overlap between orthologous groups

Typically, the orthologous groups predicted by a single execution of an all-at-once approach tool are mutually exclusive. On the other hand, running OrthoReD on large numbers of genes within a dataset can result in non-mutually exclusive orthologous groups as we see based on the fact that the number of non-overlapping merged groups is substantially fewer than the number of orthologous groups predicted by OrthoReD (Table 5). This difference however does not necessarily reflect a difference in the quality of orthology predictions between an all-at-once approach and a one-at-a-time approach.

The fact that a given gene can be found in multiple orthologous groups by searching from a different gene of interest as a starting point by OrthoReD indicates that the phylogenetic signal is not sufficiently strong to eliminate all but one possibility. In such a case, separating all genes into mutually exclusive orthologous groups by all-at-once approach tools could give the false sense of confidence in its prediction.

### Future directions

We found that while the median CPU time for ReD_n4 for the 100-species containing ACTINO was only marginally longer than ReD_n10 for PLANT, containing 13 species, the upper quartile was longer in ReD_n4, reaching >70 min. This suggests that as the number of species included in the dataset is increased, the time becomes longer. One way to maintain the efficiency of OrthoReD would be to incorporate additional screening methods to reduce the number of sequences entering into the tree-building step. For example, HMMER [35] could be implemented between steps 4 and 5 as it is used in [36] to generate a gene family profile. HMMER, given a MSA and a dataset, will first make a hidden Markov model (HMM) based on the MSA, and then searches for sequences within the dataset that fit the HMM. Currently, the parameter for MCL we use is as relaxed as possible to minimize the risk of false negatives. Implementation of HMMER after MCL should allow for a more stringent parameter setting for MCL, giving a smaller cluster of interest and subsequently rescuing some genes based on the HMM. However it must be noted that HMMER searches are most effective at detecting sequences with conserved regions such as functional domains. So upon the implementation of HMMER into OrthoReD, the risk of false negatives and the benefit of increased speed need to be balanced.

## Conclusions

Without requiring a high-performance computing cluster, OrthoReD was able to generate results comparable to other published orthology prediction tools with animal (FLY), plant (PLANT), and bacterial (ACTINO) datasets that ranged in species relatedness, prevalence of gene duplication, and number of species.

Collecting orthologs is a step required in phylogenetic analyses of gene sequences. A strategy commonly employed in functional genetics is to apply orthology prediction in silico to identify candidates for functionally equivalent genes followed by in vivo confirmation (e.g. Isaacs et al. [37]). High-throughput sequencing has already become widely available, and genetic manipulation methods (e.g. Jiang et al. [38]) even for non-model organisms, are being established rapidly. As such, there is now an increasing need for identifying orthologs and/or functional equivalents across the spectrum of organisms.

Many of the automated orthology prediction tools currently available are designed to analyze the entire dataset at once. While such tools are powerful and useful for orthology predictions, for example, of an entire genome/transcriptome, large-scale analysis has some limitations for studying a more focused subset of the genome: time is spent predicting orthologies of genes that are not a part of the subset of interest. Because orthology prediction is dataset-specific, loss in time can be exacerbated each time any sequence included in the dataset is updated, requiring the orthology predictions to be repeated.

In addition to accuracy as an orthology prediction tool, OrthoReD offers flexibility: depending on the specific experimental design, OrthoReD can be executed on a single gene of interest, on a subset of genes in a genome, or on all the genes in the genome. This flexibility allowing the user to only analyze the genes of interest, in turn, reducing the time for analysis. Moreover, since OrthoReD does not require an often costly high-performance computing cluster, orthology predictions for focused portions of high-throughput sequencing data are accessible to a wide range of researchers.

## Additional files

Additional file 1: Table S1. List of genomes used for each dataset. (XLSX 55 kb)

Additional file 2: Table S2. Number of false positive and negative gene predictions of each condition against OID on PLANT. (XLSX 35 kb)

## Abbreviations

BBH: Bidirectional best hit
CDS: Coding sequences
CPU: Central processing unit
HMM: Hidden Markov model
MSA: Multiple sequence alignment
ODB: OrthoDB
OID: OrthologID

## References

[1] Fitch WM (2000). Homology: a personal view on some of the problems. Trends Genet.

[2] Altschul SF, Gish W, Miller W, Myers EW, Lipman DJ (1990). Basic local alignment search tool. J Mol Boil.

[3] Lechner M, Hernandez-Rosales M, Doerr D, Wieseke N, Thevenin A, Stoye J, Hartmann RK, Prohaska SJ, Stadler PF (2014). Orthology detection combining clustering and synteny for very large datasets. PLoS One.

[4] Arvestad L, Berglund A, Lagergren J, Sennblad B (2003). Bayesian gene/species tree reconciliation and orthology analysis using MCMC. Bioinformatics.

[5] Chiu JC, Lee EK, Egan MG, Sarkar IN, Coruzzi GM, DeSalle R (2006). OrthologID: automation of genome-scale ortholog identification within a parsimony framework. Bioinformatics.

[6] Li L, Stoeckert CJ, Roos DS (2003). OrthoMCL: identification of ortholog groups for eukaryotic genomes. Genome Res.

[7] Ebersberger I, Strauss S, Von Haeseler A (2009). HaMStR: profile hidden markov model based search for orthologs in ESTs. BMC Evol Biol.

[8] Ostlund G, Schmitt T, Forslund K, Kostler T, Messina DN, Roopra S, Frings O, Sonnhammer ELL (2010). InParanoid 7: new algorithms and tools for eukaryotic orthology analysis. Nucleic Acids Res.

[9] Altenhoff AM, Gil M, Gonnet GH, Dessimoz C (2013). Inferring hierarchical orthologous groups from orthologous gene pairs. PLoS One.

[10] Hulsen T, Huynen MA, De Vlieg J, Groenen PMA (2006). Benchmarking ortholog identification methods using functional genomics data. Genome Biol.

[11] Wolf YI, Koonin EV (2012). A tight link between orthologs and bidirectional best hits in bacterial and archaeal genomes. Genome Biol Evol..

[12] Dalquen DA, Dessimoz C (2013). Bidirectional best hits miss many orthologs in duplication-rich clades such as plants and animals. Genome Biol Evol.

[13] Yang Y, Smith SA (2014). Orthology inference in nonmodel organisms using transcriptomes and low-coverage genomes: improving accuracy and matrix occupancy for phylogenomics. Mol Biol Evol.

[14] Huerta-Cepas J, Dopazo H, Dopazo J, Gabaldon T (2007). The human phylum. Genome Biol.

[15] Huerta-Cepas J, Capella-Gutierrez S, Pryszcz LP, Marcet-Houben M, Gabaldon T (2014). PhylomeDB v4: zooming into the plurality of evolutionary histories of a genome. Nucleic Acids Res.

[16] Moreno-Hagelsieb G, Latimer K (2008). Choosing BLAST options for better detection of orthologs as reciprocal best hits. Bioinformatics.

[17] Gish W. AB-BLAST. 1996-2009. http://blast.advbiocomp.com. Accessed 8 Dec 2016.

[18] Rognes T (2011). Faster Smith-waterman database searches with inter-sequence SIMD parallelization. BMC Bioinformatics.

[19] Korf I, Yandell M, Bedell J. BLAST protocols. In: Korf I, Yandell M, Bedell J, editors. BLAST. Sebastopol: O’Reilly; 2003. p 130-158.

[20] Sander C, Schneider R (1991). Database of homology-derived protein structures and the structural meaning of sequence alignment. Proteins.

[21] Smith SA, Pease JB. Heterogeneous molecular processes among the causes of how sequence similarity scores can fail to recapitulate phylogeny. Brief Bioinform. 2017;18(3):451–457. 10.1093/bib/bbw034 PMC5429007 27103098

[22] Van Dongen S. Ph.D. thesis: Graph Clustering by Flow Simulation. Amsterdam: Stichting Mathematisch Centrum; 2000.

[23] Katoh K, Standley DM (2013). MAFFT multiple sequence alignment software version 7: improvements in performance and usability. Mol Biol Evol.

[24] Stamatakis A (2014). RAxML version 8: a tool for phylogenetic analysis and post-analysis of large phylogenies. Bioinformatics.

[25] Kuck P, Mayer C, Wagele J, Misof B (2012). Long branch effects distort maximum likelihood phylogenies in simulations despite selection of the correct model. PLoS One.

[26] Kriventseva EV, Tegenfeldt F, Petty TJ, Waterhouse RM, Simao FA, Pozdnyakov IA, Ioannidis P, Zdobnov EM (2015). OrthoDB v8: update of the hierarchical catalog of orthologs and the underlying free software. Nucleic Acids Res.

[27] OrthoDB. University of Geneva, Geneva. 2007–2016. http://www.orthodb.org/?page=downloads. Accessed 10 Oct 2016.

[28] The Angiosperm Phylogeny Group (2009). An update of the angiosperm phylogeny group classification for the orders and families of flowering plants: APG III. Bot J Linn Soc.

[29] Goodstein DM, Shu S, Howson R, Neupane R, Hayes RD, Fazo J, Mitros T, Dirks W, Hellsten U, Putnam N, Rokhsar DS (2012). Phytozome: a comparative platform for green plant genomics. Nucleic Acids Res.

[30] Phytozome. The Regents of the University of California. 1997–2015. https://phytozome.jgi.doe.gov/pz/portal.html. Accessed 10 Oct 2016.

[31] Sen A, Daubin V, Abrouk D, Gifford I, Berry AM, Normand P (2014). Phylogeny of the class *Actinobacteria* revisited in the light of complete genomes. The orders ‘*Frankiales*’ and *Micrococcales* should be split into coherent entities: proposal of *Frankiales* ord. Nov., *Geodermatophilales* ord. Nov., *Acidothermales* ord. Nov. and *Nakamurellales* ord. Nov. Int J Syst Evol Microbiol.

[32] Markowitz VM, Chen IA, Palaniappan K, Chu K, Szeto E, Pillay M, Ratner A, Huang J, Woyke T, Huntemann M, Anderson I, Billis K, Varghese N, Mavromatis K, Pati A, Ivanova NN, Kyrpides NC (2014). IMG 4 version of the integrated microbial genomes comparative analysis system. Nucleic Acids Res.

[33] IMG/M. The Regents of the University of California. 1997–2016. https://img.jgi.doe.gov. Accessed 10 Oct 2016.

[34] Rensing SA (2014). Gene duplication as a driver of plant morphogenetic evolution. Curr Opin Plant Biol.

[35] Eddy SR (1998). Profile hidden Markov models. Bioinformatics.

[36] Wickett NJ, Mirarab S, Nguyen N, Warnow T, Carpenter E, Matasci N, Ayyampalayam S, Barker MS, Burleigh JG, Gitzendanner MA, Ruhfel BR, Wafula E, Der JP, Graham SW, Mathews S, Melkonian M, Soltis DE, Soltis PS, Miles NW, Rothfels CJ, Pokorny L, Shaw AJ, DeGironimo L, Stevenson DW, Surek B, Villarreal JC, Roure B, Philippe H, DePamphilis CW, Chen T, Deyholos MK, Baucom PS, Kutchan TM, Augustin MM, Wang J, Zhang Y, Tian Z, Yan Z, Wu X, Sun X, Wong GK, Leebens-Mack J. Phylotranscriptomic analysis of the origin and early diversification of land plants. PNAS. 2014;111(45):E4859–68. 10.1073/pnas.1323926111 PMC4234587 25355905

[37] Isaacs M, Carella P, Faubert J, Rose JKC, Cameron RK (2016). Orthology analysis and *in vivo* complementation studies to elucidate the role of DIR1 during systemic acquired resistance in *Arabidopsis thaliana* and *Cucumis sativus*. Front Plant Sci.

[38] Jiang W, Zhou H, Bi H, Fromm M, Yang B, Weeks DP (2013). Demonstration of CRISPR/Cas9/sgRNA-mediated targeted gene modification in Arabidopsis, tobacco, sorghum and rice. Nucleic Acids Res.

